# Comparison of CytoSorb and Jafron HA330 Hemoadsorption Devices in Pediatric Oncological Patients with Sepsis: Retrospective Observational Study

**DOI:** 10.3390/jcm13247694

**Published:** 2024-12-17

**Authors:** Diana Ryazanova, Zaure Tobylbayeva, Olga Mironova, Erken Kakenov, Vitaliy Sazonov

**Affiliations:** 1 Department of Medicine, School of Medicine, Nazarbayev University, Astana Z05K4F4, Kazakhstan; diana.ryazanova@nu.edu.kz; 2 Pediatric Anesthesiology and Intensive Care Unit, Mother and Child Center, “University Medical Center”, Astana Z05K4F4, Kazakhstan; 3 Department of Surgery, School of Medicine, Nazarbayev University, Astana Z05K4F4, Kazakhstan

**Keywords:** hemadsorption, sepsis, pediatric sepsis, cytosorb, HA330, pediatric oncology

## Abstract

**Background:** Pediatric sepsis presents a severe risk to immunocompromised children, especially those with cancer or pre-existing conditions, posing a significant threat to their lives. Cytokine hemadsorption has emerged as a promising therapeutic approach for managing sepsis and severe inflammatory conditions in critically ill patients. This innovative method involves eliminating pro-inflammatory cytokines from the bloodstream, targeting the underlying hyper-inflammatory response often seen in critical illnesses. Study aim: The study aim is to examine and compare the efficacy of HA330 and CytoSorb for extracorporeal blood purification in septic children with oncology. **Methods:** In this retrospective observational study, we examine 20 cases to assess the effectiveness of hemoperfusion therapy using hemoadsorption devices in pediatric septic patients with oncology. Our focus is on the use of HA330 and Cytosorb hemoadsorption devices, both designed to remove bacterial toxins and inflammatory agents from the bloodstream. **Results:** Our study reveals that hemoadsorption with HA330 and CytoSorb effectively treats septic children with oncological conditions. **Conclusions:** The presented findings suggest no statistically significant difference between the two devices in reducing the levels of the assessed parameters for extracorporeal blood purification in this patient population.

## 1. Introduction

Cancer stands as the second leading cause of death among children aged 1 to 14 years, surpassed solely by accidents. Among childhood cancers, leukemia takes the forefront, accounting for 28% of cases, closely followed by brain and other nervous system tumors at 27% [1]. Recent studies show that despite significant advances in modern medicine, the mortality rate associated with sepsis and septic shock remains alarmingly high, reaching up to 50% [2]. This underscores sepsis as a serious global public health challenge and highlights the urgent need for improved therapeutic strategies and interventions.

Sepsis is a severe and life-threatening condition that poses a significant threat to immunosuppressed children, particularly those with cancer or prior diseases. The immunosuppressive effects of cancer and chemotherapy, coupled with the challenges of late identification and hospital environments, make it essential for healthcare professionals to be vigilant in monitoring and managing sepsis in this vulnerable population [3,4]. Early recognition and prompt intervention are crucial in improving the prognosis and reducing the high mortality associated with sepsis in immunosuppressed children [5]. The prognosis for severe sepsis is notably grim. While currently relied upon, physiological parameters may eventually be supplanted by more objective and reliable biochemical markers of inflammation, such as elevated C-reactive protein and IL-6 levels [6].

When conventional therapy is insufficient, hemadsorption, particularly cytokine hemadsorption, has emerged as a valuable treatment option for managing sepsis and severe inflammatory conditions in critically ill patients. This innovative approach involves the removal of pro-inflammatory cytokines from the bloodstream, addressing the underlying hyper-inflammatory response that often accompanies critical illnesses [7,8].

There are two different approaches to hemoperfusion for sepsis treatment. One involves selectively targeting an endotoxin. The other is more comprehensive, focusing on general adsorption, although it has yet to be extensively tested [9].

One of the selective hemadsorption devices making significant strides in this field is Jafron HA330. This hemoperfusion cartridge features a porous resin designed to eliminate cytokines, complements, and other endotoxins within the molecular weight range of 10–60 kDa [10].

CytoSorb is another selective hemadsorption technology making significant strides in critical care medicine. This device employs porous polymer beads to adsorb molecules within the 5–55 kDa range, including numerous cytokines and damage-associated molecular patterns [11]. The use of hemadsorption techniques like HA330 and CytoSorb represents a promising advancement in managing sepsis and hyper-inflammatory conditions. These technologies offer healthcare professionals means to target the underlying inflammatory cascade directly, potentially improving patient outcomes.

The aim of this study is to examine and compare the clinical efficacy of HA330 and CytoSorb for extracorporeal blood purification in septic children with oncology.

## 2. Materials and Methods

### 2.1. Study Design

This retrospective observational study aimed to compare the efficacy of extracorporeal blood purification therapy using hemoadsorption devices in pediatric patients with sepsis. We focused on utilizing the HA330 hemoadsorption device by Jafron Biomedical Co., Ltd., Zhuhai, China (HA330), and CytoSorb by CytoSorbents Corporation, Plainsboro, NJ, USA (CytoSorb), designed to remove bacterial toxins and inflammatory mediators from the bloodstream.

We retrospectively reviewed and analyzed the electronic medical records (EMRs) of pediatric patients with sepsis treated at the Tertiary Hospital in Astana, Kazakhstan, between 2021 and 2023. This study was carried out in accordance with the ethical standards of the institutional research committee and with the 1964 Helsinki Declaration and its later amendments or comparable ethical standards. This study was approved by the Institutional Research Ethics Committee and, due to its retrospective nature, the requirement for informed consent for the study was waived. All patient data were deidentified to maintain confidentiality in accordance with HIPAA regulations. Prior to the procedure, signed written informed consent was obtained from all participants’ parents.

This study includes pediatric patients 17 years of age or younger diagnosed with any oncologic pathology. Eligible participants were those admitted to the Pediatric Intensive Care Unit (PICU) with a diagnosis of sepsis and/or septic shock, multi-organ failure, or cytokine storm. Patients must have failed to improve with conventional therapy and have subsequently experienced a worsening of their condition. Inclusion required indications for renal replacement therapy. All patients underwent hemosorption with either the CytoSorb or Jafron HA330 device. This study did not restrict inclusion based on treatment outcome. Documentation of leukocyte count, CRP, PCT, and IL-6 levels measured before and after the hemosorption procedure was required for inclusion.

The choice of these biomarkers considers the varying laboratory capabilities in different clinical settings. Recognizing that these capabilities may vary, the use of these well-established indicators ensures that the results are reliable and consistent regardless of minor variations in laboratory performance. Second, these parameters align our study with the existing literature, facilitating comparability with previous and future research in the field. This standardization is critical for meta-analyses and systematic reviews, increasing this study’s relevance and utility in broader clinical contexts. Finally, these biomarkers are proven indicators of inflammatory response and organ dysfunction, making them effective measures for assessing the therapeutic impact of hemosorption in patients with sepsis and septic shock. By selecting these indicators, we aim to provide a robust evaluation of treatment efficacy and contribute valuable insights into the management of critical conditions in pediatric oncology patients. No control group was included, as the study focused on comparing the efficacy and safety of the two hemoadsorption devices.

At the same time, we analyzed general epidemiological data (age, gender, weight, primary diagnosis) and clinical status (oxygenation index, inotropic support, presence of renal, and other organ damage according to the pSOFA score). Efficacy was assessed by the degree of reduction in inflammatory markers, reduction in the need for external support (mechanical ventilation, intravenous support, renal replacement therapy), and reduction in the pSOFA score.

### 2.2. Intervention

Currently, there is no consensus and no universal parameters that would serve as an indication for hemoperfusion in children. In our case, the decision to perform hemoperfusion was made by a team of physicians, including intensivists, oncologists, and nephrologists. In most cases, we encountered a so-called cytokine storm, combined with sepsis and developing multiorgan injury. The parameters that signal the start the extracorporeal cytokine absorption therapy were as follows: interleukin 6 levels—above 43.0 pg/mL, PCT levels—above 0.1 ng/mL, and CRP levels—above 1.0 mg/mL. Based on our experience, we favor the early initiation of hemoperfusion, within the first 12 h after the diagnosis of septic shock and the development of a cytokine storm.

All patients were presenting with acute kidney injury and underwent initiation of pediatric continuous venovenous hemodiafiltration (CVVHDF) using the “Prismaflex” device (Baxter, Deerfield, IL, USA) with a standard hemofilter (Polyarylethersulphone or ANST69). The prescribed parameters for this procedure included a blood flow rate of 5–10 mL/kg/min, both pre-filter reinfusion and an effluent dose of 2000 mL/h/1.73 m^2^, an ultrafiltration rate of 2–5 mL/kg/h, prolonged heparinization ranging from 5 to 50 IU/kg/h, an effluent rate of 60–100 mL/kg/h, and a dialysate fluid rate of 30–50 mL/kg/h. The disposable hemoperfusion cartridge HA330 was utilized for a duration of 4 h for the respective group. Treatment with the CytoSorb cartridge lasted on average for 24 h.

A suitable hemodialysis catheter, proportional to the child’s size, was inserted into the right or left subclavian vein. Before connecting the device to the patient, normal saline was employed for priming the circuit and system testing, which was replaced with red blood cell suspension if needed.

The utilization of hemoadsorbers was terminated following the designated time frame for each device—four hours for HA330 and 24 h for CytoSorb. This decision stemmed from the notable decline in hemoadsorber efficacy with prolonged usage, rendering it ineffectual. In instances when patients required extended hemodialysis, the hemoadsorbers were extracted from the circuit, allowing hemodialysis to persist independently. Furthermore, while cytokine levels in the bloodstream exhibited a reduction during treatment, clinically significant alterations manifested only after 12–16 h post-procedure. Consequently, providing precise parameter values to warrant the discontinuation of HA330 or CytoSorb therapy beyond manufacturer guidelines proves futile.

### 2.3. Statistical Analysis

Statistical analyses were performed with Stata version 16.1. Continuous variables were tested for normality using the Shapiro–Wilk test. Based on their distributions, variables were described as means ± standard deviations for normally distributed data or as medians and interquartile ranges for non-normally distributed data.

Comparisons between the two device groups (HA330 and CytoSorb) regarding the reduction in inflammatory markers (leukocytes, CRP, procalcitonin and IL-6) were performed using independent sample *t*-tests for normally distributed variables. Levene’s test for equality of variances was performed to test the assumption of homogeneity of variance before performing the *t*-tests.

Outliers were identified using the interquartile range method. Identified outliers were evaluated and discussed to determine their impact on the analysis, and decisions regarding their inclusion were based on their potential to bias the results. Outliers with clinically implausible values or data entry errors were excluded from the analysis. All tests were two-tailed, and a *p*-value of less than 0.05 was considered statistically significant. The results are presented with the corresponding 95% confidence intervals to provide a range within which the true values of the estimates are likely to fall. Power analysis was not performed due to the retrospective nature and small size of this study.

## 3. Results

After analyzing the medical records for the specified period, twenty patients were included in this study. The patients were divided into two groups based on the device used: the HA330 group (*n* = 10) and the CytoSorb group (*n* = 10). The decision to use a particular device depended solely on its availability in the clinic at a given time.

The children included in this study presented with various diagnoses, such as pure red cell aplasia, drug-resistant relapsing acute lymphoblastic leukemia (ALL), sarcoma, and others, with ages ranging from 6 months to 14 years old. The distribution of different diagnoses, as well as age, gender, and body mass for each patient in both groups, is shown in Table 1. In the HA330 group, there were six females and four males, while in the CytoSorb group, there were five females and five males.

All cases treated with both HA330 and CytoSorb devices showed a positive response to the therapy, as indicated by improvements in inflammatory markers (as outlined in Table 2A) and clinical parameters (Table 2B). Given the potential efficacy of standard filters against cytokines, we included a comparison group of septic patients with similar clinical profiles to evaluate the efficacy of the adsorbers. In this group, only renal replacement therapy sessions were performed without the integration of hemosorption. However, this group cannot be considered a pure control group for several reasons. The most important of these are the lack of oncological diagnoses, non-identical conditions, and variations in the timing of initiation of replacement therapy. The inability to establish a pure control group is due to the limited number of cases with identical clinical conditions.

A total of 11 procedures were conducted (with one patient undergoing the procedure twice) for the HA group and 10 for the CytoSorb group, during which we observed a significant decrease in all inflammatory markers. At the same time, no significant decrease in platelet count was observed in our study; rather, in some cases, a slight increase was observed. This phenomenon may be due to the effects of ultrafiltration and continuous kidney replacement therapy in general. In addition, it is important to note that CVVHDF, even in the absence of hemoadsorption, can moderately reduce inflammatory markers. However, a clinically significant effect is only observed when these therapies are used in combination.

Conventional treatment and antibiotic therapy appeared insufficient in these patients, but the use of disposable hemoperfusion cartridges seemed to play a crucial role in stabilizing their clinical condition. Regardless of the hemoadsorber used, we clinically observed a decrease in the pSOFA scale, a decrease or complete withdrawal of inotropic support, and improved oxygenation. The onset of clinical improvement was observed 12–16 h after the procedure. However, more significant milestones, such as complete weaning from mechanical ventilation or complete withdrawal of inotropic support, were typically achieved later, on average between 48 and 72 h after the procedure. In terms of long-term outcomes, four deaths were noted; all were due to the progression of the underlying oncologic disease rather than the treatment interventions.

It is important to note that the mean and standard deviation are among the simplest and most visual methods of presenting results. Based only on them, it is possible to observe that both devices show similar reduction rates. Despite this, all the results have a substantial standard deviation (in one of the cases, the standard deviation is even greater than the mean). Frankly, parameter results are expected to be scattered, given the nature of these data. An independent sample *t*-test was carried out for a more in-depth comparison between paired parameters (ex., leukocytes of the HA330 group vs. CytoSorb). The data obtained using SPSS are presented in Table 3.

As can be seen from the calculation results, the two-sided *p*-value for all four parameters is greater than 0.05, which means that the difference between the reduction rates of the two devices is statistically insignificant. Simply put, they performed almost identically, and the difference between the effects is minor.

Two statistical outliers were also noticed in the data. Patient 2 from the HA330 group had a leukocyte concentration of 0.01 × 109/L, which, after therapy with the device, became 0.03 × 109/L, making the reduction rate +200%. First of all, this patient initially had an insufficient white blood cell count, and further reduction was not a function of this device. Also, this number is positive (that is, it is not a reduction but an increase rate) and can be interpreted as an incorrect representation of the device’s performance.

A second outlier was also seen in the HA330 group, where Patient 9 had a clinically acceptable white blood cell count of 15.1 × 109/L, which after therapy became 14.98 × 109/L, making the reduction rate < −1%. Such a minimal decrease in the already normal level of leukocytes does not portray the effectiveness of the HA330 device.

Based on the above observations, these data were removed from the calculations in an attempt to make a more explicit analysis. The results of the new independent sample *t*-test for leukocytes are presented in Table 4.

Despite the change in the mean and the decrease in the standard deviation (indicating a smaller scatter of data), the two-sided *p*-value for leukocytes is still greater than 0.05, which again makes a difference between the performances of these two devices statistically insignificant.

## 4. Discussion

Our study endeavors to address this critical intersection of health challenges by seeking optimal strategies for alleviating the burden of sepsis in pediatric oncology patients. In this study, we aimed to compare the efficacy of two hemoadsorption devices in reducing the levels of four medical test parameters in pediatric patients with oncological diagnoses. Based on the reduction rates of these parameters, it will be possible to evaluate and compare the effectiveness of both devices.

Our statistical analysis revealed that the observed difference in efficacy between the two devices was statistically insignificant. These findings have several important implications and considerations.

The lack of significant difference in efficacy between the two hemoadsorption devices suggests that both devices perform similarly in terms of reducing the levels of the tested parameters. This finding may indicate that the mechanisms of action or the design characteristics of the two devices are comparable in their ability to remove the targeted substances from the bloodstream.

An interesting point arose when considering the design of both devices. The molecular weight of IL-6 is around 21 kDa, and PCT’s weight is about 13 kDa [12], and the CRP is considered to be around 120 kDa [13], while leukocytes’ weight can vary depending on factors such as the specific subtype of the leukocyte (e.g., neutrophils, lymphocytes, monocytes) and their activation status. Nevertheless, the weights of both molecules exceed the capabilities of the studied devices. Therefore, their decrease is not attributed to the sorption capabilities of these devices. Hence, we assume that the drop in levels of leukocytes and CRP results from decreased levels of IL-6 and PCT.

Investigating potential biomarkers, IL-6 and C-reactive protein (CRP) were scrutinized, alongside PCT, which emerged as another biomarker due to its elevation in bacterial infections [14]. While IL-6’s prognostic value in sepsis is well documented, its diagnostic specificity is limited [15]. Conversely, CRP’s significant rise during acute inflammation has made it a longstanding marker for inflammatory or infectious diseases, particularly in pediatric cases [16].

Studies initially demonstrated PCT’s superiority over CRP in bacterial infection diagnosis, with recent research suggesting its ability to predict blood culture results in critically ill patients. In patients with septic shock, analyzing white blood cell count reveals distinct groups with rising trajectories linked to higher mortality rates [17].

From a clinical perspective, the similar efficacy of the two devices in lowering these parameters is reassuring, as it suggests that healthcare providers may have flexibility in choosing between them based on other factors such as cost, availability, or availability of research. For example, HA330 treatment lasts 4 h compared to CytoSorb’s 24 h procedure, leading to a similar outcome.

As noted above, significant clinical improvement typically occurs after approximately 48–72 h, which is somewhat faster than has been reported in adult populations [18]. This earlier improvement in children may be due to factors such as fewer comorbidities, more robust and adaptive immune responses, and different metabolic profiles—all of which may facilitate a more rapid return to homeostasis compared to adults.

Both devices necessitate approximately 300 mL of blood to fill the hemoadsorber. This extended filling process not only prolongs the duration of the CVVHDF circuit but also entails withdrawing a relatively high volume of blood. Exploring the potential of the HA60 device, which requires only 65 mL of blood, holds promise for improving acceptability, particularly for newborns and children. The use of CytoSorb 150 mL in hemadsorption led to positive results for two patients experiencing multi-system organ failure after refractory septic shock and acute liver failure. The adsorber column of 150 mL still required a significant portion of the circulating blood volume for an infant. Newborns and neonates would benefit from employing a smaller cartridge column with a reduced loading volume for wider application of this treatment [19].

Presently, CytoSorb is available with a loading volume of 150 mL and 300 mL, the latter aligning with HA300. This similarity renders them comparable for our study. However, the HA series’ innovation of a lower loading volume of 60 mL represents a notable advantage for infants and neonates. Another notable advantage of HA330 is its lower price point, which makes it the preferred option for the hospitals in low-income countries. Conversely, the higher price of CytoSorb restricts its availability in these settings.

In addition to the above, Jafron offers other adsorbers that could potentially be used in pediatric intensive care patients with oncology. For example, a recent case report demonstrated the efficacy and safety of hemosorption with HA230 for the impaired elimination of methotrexate after high-dose chemotherapy in a patient with AKI [20].

A recent study recommends starting therapy with the HA330 device as soon as possible—within 48 h of diagnosing sepsis. Although this is an adjuvant therapy, the findings of their study show a shorter stay in the PICU for patients who underwent this therapy within the first 48 h—their average stay in the PICU was approximately 14 days, compared to those who began treatment after 48 h—46 days on average [21].

The observation that CytoSorb has garnered more attention in pediatric studies compared to HA330 is significant, particularly when considering its FDA approval status [22]. The abundance of research dedicated to CytoSorb’s pediatric applications underscores its established safety profile and efficacy in this vulnerable patient population. Conversely, the relatively limited exploration of HA330 in pediatric settings may stem from various factors, including regulatory considerations, research priorities, and funds. Whatever the case is, efforts to expand the evidence base for HA330 in pediatric populations could help address this gap and provide clinicians with additional therapeutic options to optimize patient care.

In cases of sepsis, children often experience multiple organ failure, necessitating additional supportive therapy. In our observations, children with sepsis and multiple organ failure displayed reduced diuresis. Consequently, they were connected to the CVVHDF system. A hemoadsorber was integrated into the CVVHDF circuit to enhance treatment effectiveness. The Prismaflex hemofilter employed in CVVHDF only partially removes IL-6 [23]. Furthermore, building on our prior encounter involving the Prismaflex device equipped with an AN69 filter [24], it became apparent that CVVHDF with Prismaflex alone did not sufficiently eliminate IL-6. Consequently, hemoadsorption therapy emerged as a requisite measure for mitigating the severity of sepsis. A standalone CVVHDF session is typically insufficient for eliminating these inflammatory mediators. Thus, the careful selection of cartridges can significantly impact the elimination of cytokines and subsequent improvement of patients’ health.

Another critical point is that in order to prevent a patient’s blood from coagulation, heparin was introduced into the CVVHDF circuit. Heparin is known to cause side effects. For example, a potential danger associated with the use of heparin infusions and circuits coated with heparin is the development of heparin-induced thrombocytopenia [25]. Therefore, a shorter duration of treatment by HA330 and shorter exposure to heparin is beneficial.

Nevertheless, heparin has multiple alternatives and can be replaced for the safe use of hemoadsorption devices. Regional citrate anticoagulation has the potential to notably enhance the effectiveness of continuous renal replacement therapy in severely ill patients and improve their blood coagulation parameters [26]. Due to the considerable risk of blood clot formation linked with extracorporeal devices, various methods involving antiplatelet and anticoagulant agents have been employed to reduce the likelihood of clotting within the patient and the device. These methods include unfractionated heparin, low-molecular-weight heparin, citrate, and direct oral anticoagulants [27]. Overall, patients can potentially benefit from having multiple options for hemoadsorption therapy without compromising treatment effectiveness.

Apart from CVVHDF, hemoadsorption devices can also be incorporated into the ECMO circuit. Dr. Lesbekov (2022), in his research, studied the efficacy of both devices for patients on V-A ECMO. Hemoadsorption therapy administered to patients undergoing postcardiotomy ECMO support demonstrated promising therapeutic efficacy [11].

While our study predominantly focuses on assessing the reduction in parameter levels and overall patient outcomes, it is essential to acknowledge that fatal cases exist within the spectrum of patient experiences. Retrospective papers and broader literature reviews often include analyses of fatal cases, providing valuable insights into the potential limitations and challenges of hemoadsorption therapy. Mortality is reported to be higher in groups that were not treated with the use of any hemoadsorption device [11]. By acknowledging the presence of fatal cases, we aim to avoid bias and ensure transparency in our findings. Despite reporting a relatively low mortality rate in our study, it is imperative to recognize that other studies may document higher mortality rates. These findings underscore the importance of not overstating the efficacy of the devices and acknowledging their potential limitations and variability in clinical outcomes across different patient populations and healthcare settings.

In this study, we reported the distribution of oncological conditions among selected patients. Acute lymphoblastic leukemia (ALL) emerged as the predominant condition among both groups, comprising 14 out of 20 cases. Given that a significant proportion of patients necessitating hemoadsorption treatment were diagnosed with ALL, it raises the possibility that children with ALL may be more prone to developing sepsis. This echoes a previous observation indicating a high prevalence of sepsis (11.7%) among 94 children diagnosed with ALL, with a concerning mortality rate of 45.5% [28]. Nonetheless, the underlying factors driving this association remain unexplored and require further investigation.

Despite this study’s strengths, several limitations should be acknowledged. In this study, we utilized a limited set of four parameters—leukocytes, C-reactive protein (CRP), interleukin-6 (IL-6), and procalcitonin (PCT)—to evaluate the efficacy of the hemoadsorption devices. The selection of these parameters was constrained by the available resources and capabilities of the hospital’s laboratory, which limited the range of cytokines that could be measured reliably. The combination of IL-6 and PCT can function as a highly sensitive indicator for identifying severe bacterial infections in children, offering significant value in distinguishing such infections during the initial stages [15]. While leukocytes, CRP, IL-6, and PCT are widely recognized as important markers of inflammation and infection, it is acknowledged that other cytokines and biomarkers may provide valuable insights into the effectiveness of hemoadsorption therapy. Future research with a more extensive range of cytokines could provide further insights into the comparative effectiveness of hemoadsorption devices.

It is crucial to acknowledge the lack of a universal method for assessing the effectiveness of blood purification devices in treating sepsis, particularly in pediatric patients with oncological conditions. This subset of patients presents unique challenges due to their compromised immune systems and susceptibility to infections, which can be exacerbated by treatments such as chemotherapy. Recognizing this gap, researchers from France proposed a methodology in 2022 to address this issue. Criteria to assess blood purification devices in treating sepsis include the following: patient selection based on disease severity, response to conventional treatments, determining the optimal timing and duration of therapy, evaluating treatment effects on immune mediators and clinical outcomes, and understanding potential interactions with other treatments, such as antimicrobial drugs and anticoagulants [29]. Our study partially accomplished the proposed methodology, yet a more expanded and detailed approach would be expedient for further research.

Another limitation of this study is the lack of randomization in group assignment. The allocation of patients to each hemoadsorption device was not performed randomly, but rather based on clinical and logistical considerations. This non-randomized allocation may introduce selection bias and confounding factors that could influence the observed outcomes. Randomization inherently means that certain children will be deliberately deprived of the therapy under investigation. However, this deprivation, in turn, imposes an unnecessary risk on those patients.

The sample size in this study, consisting of 10 cases per device, can be considered to be relatively small. While this sample size was sufficient to assess the statistical significance of a certain magnitude, it may have limited the statistical power to detect smaller differences in efficacy between the devices. A larger sample size, with its potential to provide greater precision and reliability in estimating the true effect size, underscores the importance of robust research methods in our field.

Another limitation stems from this study’s single-institution nature. Limiting the study to a single institution could restrict the generalizability of the results to other healthcare environments that vary in patient demographics, treatment approaches, and resource levels. Therefore, caution should be exercised when extrapolating the results to broader populations.

This study is a purely descriptive case series without a control group. Without a control group for comparison, it is challenging to attribute the observed reductions in parameter levels solely to the hemoadsorption devices. Other factors, such as concurrent treatments, disease progression, or natural variations in patient physiology, could have contributed to the observed outcomes.

Addressing these limitations will enhance the validity and reliability of future studies and provide a more profound understanding of the comparative effectiveness of hemoadsorption devices in clinical practice.

Although most of our cases are successful, clinicians must be cautious when considering hemoperfusion as a therapeutic option for infants weighing less than 10 kg. Recent studies suggest that outcomes in this population remain suboptimal, with higher rates of complications and limited clinical improvement compared to older pediatric patients [30,31]. Despite advances in hemoadsorption technology, the exact mechanisms driving these poor outcomes are not fully understood. Hypothetical explanations range from hemodynamic instability due to extracorporeal circulation to altered pharmacokinetics of critical medications to the unique immunologic profile of infants. Until more robust evidence clarifies the safety and efficacy of hemoperfusion in this vulnerable cohort, careful patient selection and rigorous monitoring are essential. This highlights the need for further research to delineate patient-specific factors and refine protocols that could improve outcomes for infants undergoing hemoadsorption therapy.

To summarize the findings regarding the advantages of each device, the HA330 boasts a lower price point, rendering it more accessible to local hospitals. Additionally, the introduction of its new modification, HA60, holds promise for pediatric applications. HA330 also requires only a 4 h session, while CytoSorb treatment lasts 24–36 h to reach (as revealed) the same result. On the other hand, CytoSorb has FDA approval and a more extensive body of research. Presently, this study has not identified any significant disparities in their efficacy, suggesting that the decision should weigh these factors. However, it is essential to acknowledge the dynamic nature of these aspects. For instance, ongoing research may enhance our understanding of HA devices, while CytoSorb might introduce further modifications. Therefore, a definitive conclusion remains vague.

## 5. Conclusions

In our study, both hemoadsorption devices demonstrated comparable efficacy in pediatric oncology patients with sepsis, allowing clinicians to make a choice based on cost, local resources, and patient-specific considerations. Initiating therapy within 48 h of diagnosis appears to be beneficial, and smaller volume cartridges (such as the HA60) may reduce blood volume requirements in very young or low-weight patients. The integration of hemoadsorption into CRRT or ECMO circuits may further improve treatment, provided that careful anticoagulation management—possibly with citrate instead of heparin—is used.

In the future, expanding the range of inflammatory markers monitored will refine patient selection and help guide therapeutic decisions. Emphasizing prevention through robust infection control measures and early detection strategies can reduce the overall burden of sepsis. The development of rigorous research methods, including randomized trials and multi-institutional collaborations, will improve the evidence base for hemoadsorption interventions, ultimately benefiting this vulnerable patient population.

## Figures and Tables

**Table 1:** The distribution of different oncological diagnoses, ages, genders, and weights in both groups and the overall distribution.

Pt#	Diagnosis	Gender, Age	Weight, kg	Hemoadsorber Applied	Number of Sessions	Outcome (After Procedure)
1	Pure red cells aplasia	F, 6 mo	6.2	HA-330	2	discharged on day 14
2	Drug-resistant relapsing ALL	M, 14 yo	24	HA-330	1	discharged on day 12
3	ALL	F, 2.5 yo	12	HA-330	1	fatal outcome on day 28
4	Sarcoma	M, 10 yo	28	HA-330	1	discharged on day 12
5	ALL	F, 7 yo	21	HA-330	1	discharged on day 16
6	ALL	F, 5 yo	18	HA-330	1	discharged on day 10
7	Sarcoma	M, 14 yo	36	HA-330	1	discharged on day 16
8	AML	F, 6 yo	20	HA-330	1	discharged on day 12
9	ALL	F, 11 yo	28	HA-330	1	discharged on day 14
10	ALL	M, 3 yo	15	HA-330	1	discharged on day 19
11	ALL	F, 5 yo	18	Cytosorb	1	discharged on day 18
12	Osteosarcoma	M, 12 yo	24	Cytosorb	1	discharged on day 13
13	ALL	M, 6 yo	17	Cytosorb	1	fatal outcome on day 21
14	ALL	F, 11 yo	28	Cytosorb	1	discharged on day 15
15	Meduloblastoma	M, 12 yo	23	Cytosorb	1	fatal outcome on day 29
16	ALL	F, 4 yo	15	Cytosorb	1	fatal outcome on day 18
17	ALL	M, 9 yo	22	Cytosorb	1	discharged on day 19
18	ALL	F, 5 yo	16	Cytosorb	1	discharged on day 16
19	ALL	F, 10 yo	22	Cytosorb	1	discharged on day 11
20	ALL	M, 7 yo	21	Cytosorb	1	discharged on day 20

ALL—acute lymphoblastic leukemia, AML—acute myeloid leukemia, F—female, M—male, discharged—discharged from the PICU.

**Table 2:** (**A**) Plasma concentrations of leukocytes, CRP, procalcitonin, and IL-6 and thrombocytes before and after blood purification procedure with HA330 (mean ± SD, *n* = 11) and CytoSorb (mean ± SD, *n* = 10) and in the group with CKRT only (mean ± SD, *n* = 10). (**B**) Clinical parameters before and after blood purification procedure with HA330 (mean ± SD, *n* = 11) and CytoSorb (mean ± SD, *n* = 10).

(A)													
Parameter	HA330					CytoSorb				CKRT (CVVHDF)			
	Before		After	% Reduction		Before	After		% Reduction	Before		After	% Reduction
Leukocytes, 10^9^/L	20.15 ± 8.96		9.04 ± 5.48	−28.59 ± 79.98		17.42 ± 7.72	8.85 ± 4.36		−47.68 ± 16.62	22.1 ± 4.28		19.9 ± 4.53	−8.3 ± 11.9
CRP, mg/mL	288.17 ± 99.34		105.63 ± 49.32	−60.596 ± 19.51		242.61 ± 72.53	84.63 ± 35.561		−65.22 ± 11.24	221.2 ± 36.32		203.1 ± 38.4	−9.52 ± 5.28
Procalcitonin, ng/mL	245.10 ± 115.73		11.89 ± 13.18	−73.06 ± 23.79		36.26 ± 14.38	10.27 ± 3.61		−70.52 ± 6.02	35.7 ± 9.9		31.4 ± 10.3	−15.8 ± 7.9
IL-6, pg/mL	539.89 ± 475.02		107.7 ± 52.21	−68.02 ± 19.78		322.67 ± 54.48	111.45 ± 21.45		−64.22 ± 9.85	265.7 ± 33.76		238.2 ± 37.97	−12.27 ± 6.58
Trombocytes, 10^9^/L	138.8 ± 59.78		139.3 ± 58.53	−0.5 ± 6.46		131.4 ± 54.62	130.3 ± 61.02		1.1 ± 7.32	155.9 ± 49.3		152 ± 48.32	2.64 ± 3.52
**(B)**													
		**HA330**						**CytoSorb**					
**Parameter**		**Before**			**After**			**Before**			**After**		
SpO_2_/FiO_2_ index		204.6 ± 42.89			406.7 ± 61.51			219.3 ± 33.54			411.6 ± 23.282		
Inotropes (Norepinephrine, μg/kg/min)		0.83 ± 0.6			0.1 ± 0.1			0.95 ± 0.65			0.1 ± 0.1		
Diuresis (mL/kg/h)		0.46 ± 0.29			2.71 ± 0.26			0.48 ± 0.22			2.6 ± 0.30		
Pediatric SOFA score		16.6 ± 3.16			3.5 ± 1.17			16.7 ± 2.49			3.4 ± 1.26		

CRP—C-reactive protein, IL-6—interleukin 6, CKRT (CVVHDF)—continuous venovenous hemodiafiltration (CVVHDF) only.

**Table 3:** Two-sided *p*-value obtained via the independent sample t-test for the reduction rates of both devices.

Parameter	Reduction Rate (Mean ± SD)		Two-Sided *p* Value
	HA330 (*n* = 11)	CytoSorb (*n* = 10)	
Leukocytes, 10^9^/L	−28.59 ± 79.98	−47.68 ± 16.62	0.469
CRP, mg/mL	−60.596 ± 19.51	−65.22 ± 11.24	0.520
Procalcitonin, ng/mL	−73.06 ± 23.79	−70.52 ± 6.02	0.748
IL-6, pg/mL	−68.02 ± 19.78	−64.22 ± 9.85	0.591

CRP—C-reactive protein, IL-6—interleukin 6.

**Table 4:** Two-sided *p*-value obtained via the independent sample *t*-test for reduction rates of both devices.

	Reduction Rate (Mean ± SD)		Two-Sided *p* Value
Parameter	HA330	CytoSorb (*n* = 10)	
Leukocytes	−57.08 ± 21.35 (*n* = 9)	−47.68 ± 16.62	0.297
CRP	−60.596 ± 19.51 (*n* = 11)	−65.22 ± 11.24	0.520
Procalcitonin	−73.06 ± 23.79 (*n* = 11)	−70.52 ± 6.02	0.748
IL-6	−68.02 ± 19.78 (*n* = 11)	−64.22 ± 9.85	0.591

CRP—C-reactive protein, IL-6—interleukin 6.

## Data Availability

The data that support the findings of this study are not publicly available due to their containing information that could compromise the privacy of research participants, but are available from the corresponding author VS (by email: vitaliy.sazonov@nu.edu.kz) upon reasonable request.
